# What Is so Positive about Positive Animal Welfare?—A Critical Review of the Literature

**DOI:** 10.3390/ani9100783

**Published:** 2019-10-11

**Authors:** Alistair B. Lawrence, Belinda Vigors, Peter Sandøe

**Affiliations:** 1 Scotland’s Rural College (SRUC), West Mains Road, Edinburgh EH9 3RG, UK; belinda.vigors@sruc.ac.uk; 2 Roslin Institute, University of Edinburgh, Penicuik EH25 9RG, UK; 3 Department of Food and Resource Economics, University of Copenhagen, 1958 Frederiksberg C, Denmark; pes@sund.ku.dk; 4 Department of Veterinary and Animal Sciences, University of Copenhagen, 1870 Frederiksberg C, Denmark

**Keywords:** positive animal welfare, critical review, positive emotions, positive affective engagement, quality of life, happiness

## Abstract

**Simple Summary:**

Positive animal welfare (PAW) is thought to have come about as a response to there being too much of a focus on avoiding negatives in animal welfare science. However, despite its development over the last 10 years, it is not clear what it adds to the study of animal welfare. To clarify this, we conduct a review of the literature on PAW. We aim to identify the characteristic features of PAW and to show how PAW connects to the wider literature on animal welfare. We find that the PAW literature is characterised by four features: (1) *positive emotions* which highlights the capacity of animals to experience positive emotions; (2) *positive affective engagement* which seeks to create a link between positive emotions and behaviours animals are motivated to engage in; (3) *quality of life* which acts to give PAW a role in defining an appropriate balance of positives over negatives and; (4) *happiness* which brings a full life perspective to PAW. While the first two are already well situated in animal welfare studies the two last points open research agendas about aggregation of different aspects of PAW and how earlier experiences affect animals’ ability to have well-rounded lives.

**Abstract:**

It is claimed that positive animal welfare (PAW) developed over the last decade in reaction to animal welfare focusing too much on avoiding negatives. However, it remains unclear what PAW adds to the animal welfare literature and to what extent its ideas are new. Through a critical review of the PAW literature, we aim to separate different aspects of PAW and situate it in relation to the traditional animal welfare literature. We find that the core PAW literature is small (*n* = 10 papers) but links to wider areas of current research interest. The PAW literature is defined by four features: (1) *positive emotions* which is arguably the most widely acknowledged; (2) *positive affective engagement* which serves to functionally link positive emotions to goal-directed behavior; (3) *quality of life* which serves to situate PAW within the context of finding the right balance of positives over negatives; (4) *happiness* which brings a full life perspective to PAW. While the two first points are already part of welfare research going back decades, the two latter points could be linked to more recent research agendas concerning aggregation and how specific events may affect the ability of animals to make the best of their lives.

## 1. Introduction

Positive animal welfare (PAW) is often described as a recent idea or concept. The first formal reference to PAW appears to be in Boissy et al. [1] which was followed a year later by the first conceptual development of the concept [2]. PAW has been described as a reaction against an undue focus on negative aspects of welfare and reduction of harms (e.g., [2,3,4]). PAW has also been linked to criticism of the Five Freedoms as being too focused on negatives and harms [3,5,6]. However, it is also clear that PAW emerged from wider animal-welfare thinking which was becoming increasingly interested in positive aspects of welfare including positive emotions [1] and providing animals with resources to facilitate positive welfare [7] (see also [2]).

Given that approximately a decade has passed since the PAW concept emerged this seems an appropriate time to better understand exactly what is being added by PAW and to what extent and how the PAW concept links to the wider animal welfare literature. In this paper we will analyse the existing PAW literature to define the key features of PAW coming out of this, and their relationship with the wider animal-welfare literature. For this we have critically reviewed the literature identifying both core PAW writings that clearly contribute to development of the PAW concept, and also work in the wider literature representing relevant areas of research that are clearly linked to PAW. We have reviewed and analysed the core PAW literature to distill four features that currently define how the PAW concept is understood. We have also reviewed how these features of PAW relate to the wider literature drawing out the extent to which PAW is continuous or distinct from the wider animal welfare literature. Thus we aim to make clearer what research areas have contributed to the development of PAW and what PAW uniquely contributes both scientifically and more widely to the debate over animal welfare.

## 2. Materials and Methods

To review the literature on PAW we carried out a search using Scopus including published work up to 1 March 2019, with the terms “positive welfare” and “animal” in the authors’ keywords. This resulted in a total of 12 papers including book chapters. We then repeated the search using the same terms but now including the works’ title, abstract and author’s keywords. This added an additional 59 works. We then reviewed these 71 works for relevance ending with a total of 38. Of these 38 we found that a number of works made only passing reference to PAW sometimes with vague or no definitions of what was meant by the term. Therefore, we excluded these works thus leaving what we refer to as the core PAW literature which came to 10 papers and book chapters (Table 1a). Our search and selection strategy results in 5 of these 10 core PAW papers being the work of Mellor and colleagues.

We have also included in Table 1b a list of what we refer to as key linking papers. To be included in this list, a paper had to provide a substantial link between one of the 4 features of PAW that we identified from the core PAW literature and the wider scientific literature. Work in this list did not need to refer to PAW or to develop the PAW concept. We did not seek to provide a comprehensive list of such linking papers and in that sense we accept that our choice can be seen as subjective. However, we would argue that it is beyond the purpose of this paper to provide comprehensive literature reviews for the wider literature that relates to the 4 PAW elements we have identified.

## 3. The Defining Features of Positive Animal Welfare (PAW)

Our search for papers that reference “positive welfare” and “animal” which also clearly contribute to the development of the concept reveals a small core PAW literature of 10 papers and book chapters (Table 1a). Our subsequent content analysis of this work revealed 4 key defining features—positive emotions, positive affective engagement, quality of life and happiness—that we argue define PAW as currently discussed in the literature. In the following sections we review each of these in turn and how they relate to the wider animal welfare literature.

### 3.1. Positive Emotions

In line with Boissy et al. and de Vere and Kuczaj [1,8], we will use the term emotion as an overarching term to cover subjective experiences in animals, mainly because we believe that in animals the distinctions between terms such as emotions, affect, feelings and subjective experiences are somewhat arbitrary and not currently open to empirical testing.

The core literature is clear that one of the defining features of the PAW concept is that animals have the capacity for experiencing positive emotions: e.g.,

“However, preventing negative welfare in animals is not the same as providing them with opportunities to experience positive emotions and positive welfare”.

*([4] p. 81)*

“As with negative aspects of welfare, the opportunity for animals to have positive experiences, which we describe as positive welfare”.

*([9], p. 586)*

“The increasing importance assigned to the affective (i.e., emotional) states that animals may experience and the associated greater emphasis given to the promotion of positive affective states”.

*([10], p. 1).*

The centrality of positive emotions to PAW seems to be linked to the accumulating evidence that animals can experience positive emotions, coming from different scientific areas including animal behaviour, psychology, neuroscience and animal welfare science.

*“This paper presents a rationale that may significantly boost the drive to promote positive welfare states in animals….based largely, but not exclusively, on an experimentally supported neuropsychological understanding of relationships between emotions and behaviour, an understanding that has not yet been incorporated into animal welfare science thinking”.*

*([10], p. 1)*

*“There has been a growing interest in the study of emotions in animals over the last few decades, resulting in the emergence of a discipline referred to as Affective Neuroscience (Panksepp, 1998). Scientists have made huge progress in understanding how animals perceive their environment and the feelings prompted by this perception”.*

*([4], p. 83)*

The idea that animals can experience emotions is also linked to a growing interest in animal welfare science over how we conceptualise and assess animal emotions; for example the merits of discrete emotions versus dimensional approaches consisting of core affective characteristics (e.g., valence and arousal) [11]. Given the significant drive to use animal-based measures in on-farm welfare assessment there is also considerable interest in the development of reliable and valid approaches to assess positive emotions under practical conditions (e.g., [12]).

The PAW literature has finally seen the use of terms to describe emotions that might be thought to have previously been largely applied to humans only such as pleasure, enjoyment, fun, excitement. As examples:

“Indeed, what use is there in satisfying an animal’s vital needs, if the life the animal then lives is devoid of any enjoyment?.

*([1], p. 298)*

“Negative affects are therefore incompatible with the having fun affect”.

*([13], p. 12)*

“By analogy with human beings, emotional experiences of environmentally engaged aliveness, positive excitement, even euphoria, are considered likely to attend the operation of the ‘Seeking’ system”.

*([10], p. 4)*

In summary, reference to positive emotions is a key defining feature of the PAW literature; both in terms of emphasising the capacity of animals to experience positive emotions and through the use of terms associated with positive emotion. Potentially, the use of more positive emotion terms in the PAW literature may be reflective of PAW being a reaction to an over-focus on negative aspects of welfare in the wider animal welfare literature. However, as we will describe in the following section, positive emotion in the context of animal welfare is not unique to the PAW litearature.

#### Positive Emotions: PAW Contrasted to the Wider Literature

Animal emotions have long been seen as a key aspect of animal welfare (e.g., [14]) and the focus on emotions as an element of PAW is consistent with the wider animal welfare literature. However, the emphasis on and the call for positive emotions, seen in the PAW literature, disguises the fact that there has been recognition of positive emotions in animals from the earliest origins of animal welfare concerns; for example in the Brambell Report:

“We accept that animals can experience emotions such as rage, fear, apprehension, frustration and pleasure, though they do display different degrees and types of intelligence which may affect the reaction to particular stress-causing circumstances.”.

*([15], p. 10)*

The recognition of positive emotions can also be found in scientific writing that predates PAW (e.g., [16]) and indeed many of the approaches that are proposed to assess positive emotions are the same as the methods previously proposed to measure negative emotions. For example in preference testing, whilst avoidance of a stimulus can be taken as an indication of a negative emotional response, approach behaviour can be taken to indicate a positive emotional response (e.g., [17]). In judgement or cognitive bias testing the animals’ response to ambiguous stimuli can be taken to indicate either negative or positive emotional states (e.g., [18]). Similarly in qualitative behavioural assessment (QBA) the method derives assessments of animals’ emotional (subjective) state based on human observer assessments that range from negative to positive (e.g., [19]). Play behaviour which is often proposed as a measure of positive emotional state [1] can, in some instances, be argued to result in part from negative states [20]. Perhaps the least ambiguous of all proposed measures of positive emotional state are the so-called ‘50 kHz’ ultrasonic vocalisations (USVs) produced by rats, but even here the form of these USVs may reflect the balance of positive and negative emotions being experienced [21]. In summary, all current approaches proposed to assess positive emotions in animals emphasise the continuity between PAW and the wider welfare literature.

In addition to the idea of positive emotions predating PAW, any claims of a seminal breakthrough in understanding the nature of positive emotions in animals seem to us to be exaggerated. There remains considerable scientific uncertainty about emotions in animals and, perhaps, particularly positive emotions given the relative lack of research on these [4]. For example we lack a ‘gold standard’ against which to assess and validate emotional state (negative or positive) in animals. It has been suggested that neuroscience provides the validatory evidence for positive emotional states in animals [10,22]. However, this is to ignore that there remains considerable debate within neuroscience itself over the nature of animal emotions including whether animals consciously experience emotions (e.g., [23]) and the typology best used in emotional theory [24]. For us, neuroscience provides interesting and corroborating evidence for positive emotional states in animals but it falls short of being the gold standard validating evidence. Finally, as with the assessment of emotions in general there is a risk of circularity in the assessment of positive emotions, for example where animals are exposed to a putatively positive context and the animals’ responses are then labelled as indicators of a positive state without independent validation (e.g., [25]).

As noted above, the PAW literature has seen the use of terms such as pleasure, fun and enjoyment to describe emotional states in animals that might be thought to have been largely restricted to humans. Our analysis of the use of these terms in the PAW literature is that they appear sporadically and are largely undefined. It is also not the case that terms such as pleasure and fun are only to be found in the PAW literature suggesting that PAW is not the essential trigger to the use of these terms. Darwin’s famous early account of the emotional expressions of man and animals [26] makes several references to joy and pleasure in animals:

“Under a transport of joy or of vivid pleasure we see this in the bounding and barking of a dog when going out to walk with his master; and in the frisking of a horse when turned out into an open field”.

*([26], p. 76)*

Much later, and following the development of animal behaviour science, scientific work on play in animals has often referred to the ‘enjoyment’ associated with the behaviour. For example:

“It is difficult, and probably unnecessary, to avoid applying anthropomorphic terms such as ‘spontaneous’, ‘rewarding’ and ‘enjoyable’ to the behaviour of these young animals”.

*([27], p. 508)*

Pleasure has also been used widely to describe a positive emotional state in animals, for example in affective neuroscience:

“In a sense, pleasure can be thought of as evolution’s boldest trick, serving to motivate an individual to pursue rewards necessary for fitness”.

*([28], p. 646)*

The term fun, to describe animals’ emotions, is also found in the wider literature:

“’Playing’ and ‘having fun’ are almost synonymous”.

*([29], p. 463)*

A recent special issue of the journal *Current Biology* contained a curated set of papers on fun in humans and animals including a covering editorial:

“As usual with an evolutionary question it is helpful to take a broad look at what appear to be similar behaviours in other species—in particular, to consider fun in other animals, and what functions it might have that could contribute to their evolutionary fitness”.

*([30], R1)*

Hence, perhaps contrary to expectations, the PAW literature does not represent a substantial core of writing on positive states such as joy and fun applied to animals. It would seem that PAW is essentially similar to other areas of science where recently it has become more acceptable to apply positive terms such as fun to describe animals’ state and an example of convergent evolution across science areas rather than PAW research leading the way.

Whilst we would argue that the centrality of positive emotions with respect to PAW is more of an evolution than a revolution from previous research on negative emotions, we do think it reasonable to suggest that PAW has helped bring greater attention and interest to the study of positive emotions in animals [3]. Arguably, the contribution of the PAW literature here has been to make a more explicit connection between the capacity for animals to experience positive emotions and the implications of this for their welfare.

We think it is interesting to reflect on why positive emotions are seen by some as core to the PAW concept. For example when discussing how to promote PAW, Yeates and Main [2] base their argument almost entirely on the relationships between negative and positive emotions (see p. 297), appearing to suggest that PAW and positive emotional state are more or less synonymous. This suggests that it is the growing focus, scientific and otherwise, on positive emotions that provides the justification for PAW in order to more fully recognise this capacity in animals; in effect the converse of arguing that there has been too much emphasis on negative emotional states. There may also be an influence here of the continuing interest in animal welfare science for assessing emotional states under practical (farm) conditions which is now extending to positive emotional states. It may also be that scientists are drawn to the study of positive emotions either because they themselves find reciprocal pleasure in studying animals in positive welfare states (e.g., [27]) or because they recognise the advantage of working on a subject (‘happy animals’) that the public finds engaging (e.g., [31]); for example the findings of a recent paper on the ability of goats to distinguish between positively and negatively valenced calls [32] were ‘tweeted’ 118 times and mentioned in 128 news stories [33].

Whatever the reason for the focus on positive emotions in the context of PAW we would point out the obvious, that our acceptance that animals can experience positive emotions does beg the question of how these emotions emerge in the first place and what their role is. As we present in the following section, a further element of the PAW literature—positive affective engagement—seeks to make such a functional link between goal-directed behaviours and positive emotions.

### 3.2. Positive Affective Engagement (PAE)

The need to explain how positive emotions emerge has also been a major defining feature of the PAW literature. One interpretation for the function of emotions is that they are closely linked to goal-directed behaviours. Mellor has written extensively about this in the context of PAW and coined the term positive affective engagement (PAE) which he defines as:

“…the experience animals may have when they actively respond to motivations to engage in rewarding behaviours, and it incorporates all associated affects that are positive”.

*([22] p. 3)*

PAE, as defined by Mellor [22], proposes that animal emotions are closely, if not intrinsically, linked to the guiding of goal-directed behaviours in what he refers to as the ‘emotion-motivation nexus’. In Mellor’s proposal, stimuli which are perceived as positive, produce a state of pleasure that acts as a reward and as such reinforces subsequent behaviour. The relevance of PAE for PAW is that it provides a functional link between behaviours such as foraging, maternal care and play and positive subjective experiences. In other words, it anchors positive emotions to functionally important behaviours (see also other related papers by Mellor [13,31]).

PAE, as developed by Mellor, links closely to affective neuroscience which studies how the brain generates subjective sensations including those of reward and pleasure. For example Mellor [10] uses the writings of Panksepp (e.g., [23]) who suggests that the brain has a number of discrete ‘action-orientated systems’ (including SEEKING and PLAY) that integrate emotions and motivation to organise specific behavioural responses. Mellor (e.g., [22]) also refers to the work of Berridge and co-workers (e.g., [28,34]) who have researched the neural substrates for ‘wanting’ (motivations) and ‘liking’ (sensations of pleasure) that are integral to reward systems (see also Yeates and Main [2] who discuss wanting and liking at length). Again, whilst this neuroscience research provides some corroboration for PAE we consider that there is still a considerable gap between neuroscience experimental models and the complex spontaneous behaviours (e.g., foraging, exploration, play) which are the focus of PAE.

PAE also links to other ideas on behavioural expression that have relevance to PAW. For example, Mellor makes reference to the importance of animals being able to “exercise of voluntary, self-generated behavioural expression” ([22] quoting [35]). Edgar et al. [9] base their resource-tier approach on providing animals with what they refer to as ‘good life opportunities’ specifically to allow animals the choice to engage in behaviours that will elicit positive emotions. The good life opportunities they list (comfort, pleasure, interest, confidence and health) are mainly taken from the UK’s FAWC report on a future vision for farm animal welfare [5]. In sum, PAE highlights the interconnection between behavioural expression and positive emotions and arguably situates this within PAW to draw attention to the welfare-relevance of behaviours which promote positive experiences in animals. However, as we present in the following section, the wider animal welfare literature also considers the functional link between behaviours and emotion.

#### Positive Affective Engagement: PAW Contrasted to the Wider Literature

We again see considerable continuity and overlap between PAE, with its emphasis on the intrinsic links between positive emotions, motivations and goal-directed behaviours, and the wider animal welfare literature. For example Fraser and Duncan [16] proposed the very similar concept of motivational affective states (MAS) as adaptations where subjective states are involved in motivating specific behaviours. Interestingly, Fraser and Duncan [16] make a case for negative and positive MAS serving different functions. Negative MAS (e.g., hunger; thirst) have the function of resolving needs, whereas positive MAS (e.g., play or exploration) occur to exploit opportunities when other more pressing needs are not present. The idea that negative and positive MAS might serve different functions in animals, is interestingly similar to the more recent human-based ‘broaden and build’ theory [36] which proposes that positive emotions may facilitate broadening of the mindset through play and exploration (once basic needs are met).

Other writings also propose a close link between motivation, emotions and welfare. Franks and Higgins [37] propose that in animals, like humans, well-being will be highest when animals are able to be effective in their pursuit of motivations. The importance of animals being able to express voluntary behavior [35] has been developed into the concept of animal agency [38,39,40] defined as ‘inner-motivated behavioural engagement with the environment’ which can be argued to be a key aspect of positive welfare (e.g., [39]).

In fact the continuity between PAW and the more general animal welfare literature with respect to the proposed close relationship between behavioural expression and welfare can be argued to go as far back as the Brambell Report: e.g.,

“In principle we disapprove of a degree of confinement of an animal which necessarily frustrates most of the major activities’ which make up its natural behaviour”.

*([15], p. 13)*

The emphasis on behavioural expression in the Brambell Report (including in T.H. Thorpe’s Appendix [15], p. 71) seems to have directly led to FAWC distilling the ‘Freedom to express normal behaviour’; the use of *normal* here placing emphasis on the animals’ current state rather than its previous (natural) state [41]. As a number of authors have pointed out (e.g., [2]) this freedom is not explicitly about positive welfare and more about preventing a negative (absence of frustration); yet it could equally be argued that this freedom also allows behavioural expression and associated positive emotions [42].

This early emphasis on behavioural expression and welfare was to prove an enduring focus for animal welfare research. The research that followed reasoned that normal behaviour (i.e., behaviour observed under unconstrained conditions) could be regarded as a ‘behavioural need’ which, therefore, required that housing provide the necessary ‘obligatory’ features to ‘trigger’ and satisfy the underlying motivation [43]. Following this early research, behavioural needs entered a phase dominated by analysis of the strength of underlying motivations in order to base housing requirements on the animals own priorities (e.g., the application of ‘consumer demand’ theory: [44,45]). Here the focus was on identifying the animals’ ‘necessities’ or ‘wants’ in order to minimise suffering caused by preventing strongly motivated behaviours. As pointed out by Hughes and Duncan [46], one risk of this approach is that the list of necessities as defined by motivational analysis may be insufficient to protect welfare and we would argue that this may be particularly so in the context of promoting PAW. A particular concern with respect to PAW on basing housing design and other management decisions on approaches such as consumer demand is that behaviours often regarded as potential indicators of PAW (e.g., play, exploration, positive social behaviours) may not be resilient in the presence of other stronger motivations such as hunger [47]; in the terminology of consumer demand, behaviours such as play and exploration may come to be defined as ‘luxuries’.

We would again argue that PAE, developed in the context of PAW, is a clear continuation of past conceptual work. However, it is also the case that this approach appears to have changed thinking with respect to the relationship between behavioural expression and welfare. This is particularly noticeable in the context of the debate over behavioural needs, where PAE appears to have reversed the argument back towards the original conception of behavioural needs; i.e., housing and management should facilitate normal behaviour with even an emphasis on what might have been regarded as luxury behaviours. A good example of this is the resource-tier approach [9] that aims to provide for behaviours such as curiosity driven exploration and play; behaviours that would likely not have been prioritised using a motivational analysis approach. Such explicit reference to positive experiences and their welfare relevance may be a further outcome of the PAW literature’s desire to overcome what has been seen as a narrow focus on negative aspects in animal welfare.

We would also argue that concepts such as PAE and MAS occupy a central space in PAW as they offer a link between positive emotions and the expression of evolutionary adaptive goal-directed behaviours which in turn links through to other relevant ideas and concepts. One of these is quality of life, which attempts to say something about the animals’ overall state of welfare, and where the opportunity to perform positively motivated behaviours has been highlighted as a key element of a ‘good life’ [5,9]. As we discuss in the following section, while positive emotions and PAE have focused on what contributes to the positive aspects of an animal’s life, quality of Life brings a perspective on how PAW can be used to characterise the overall welfare state of an animal.

### 3.3. Quality of Life

The quality of life (QoL) concept was effectively introduced to PAW by Yeates and Main [2] concluding that it is possible to conceive of PAW as a continuum from negative to positive:

“…the idea of a welfare continuum may be a valid model for overall welfare assessment”.

*([2], p. 297)*

As noted already, the basis of their argument is entirely based on scientific understanding of emotions and specifically the relationships between negative and positive emotions:

“Consequently, it does appear possible to describe positive and negative affect in a model that accepts the negative correlation between them, even if this is not perfect”.

*([2], p. 297)*

The FAWC (2009) report [5] followed this by conceiving of animal welfare in terms of a QoL scale, although without specifically referring to PAW:

“Our proposal, therefore, is that an animal’s quality of life can be classified as a life not worth living, a life worth living and a good life. Other classification schemes use four or more levels; three have the merit of simplicity and the basic notions are familiar in the human context”.

*([5], p. 17)*

This has proved an influential idea; currently the FAWC report [5] has been cited over 120 times, considerably more than the average for FAWC reports (Google Scholar). It has been partly responsible for triggering re-thinking over the completeness of the 5 freedoms as a welfare framework (e.g., [48]) and led to scientific-based studies based on FAWC’s QoL approach such as the resource-tier study [9]. The three-level QoL approach is now mirrored in a number of commercial farm assurance schemes (e.g., [49]).

In summary, the minimal development of QoL within the PAW literature conceives of QoL as a continuum from negative to positive with positive welfare situated at the higher end of the continuum based on either the animals’ overall emotional state [2] or the available opportunities for the animal to have a good life [5].

#### Quality of Life: PAW Contrasted to the Wider Literature

QoL is again not unique to PAW. It was originally developed in human medicine before being translated to veterinary medicine by McMillan [50]. For McMillan [50] animal QoL is defined by emotional experiences:

“Affect (subjective feelings) plays a preeminent and, I propose, exclusive role in all interpretations of QoL in animals.”.

*([50], p. 1905)*

McMillan’s conception of animal QoL, as defined by emotion, is of a continuum ranging from unpleasant to pleasant experiences, a good QoL being where pleasant experiences outweigh the unpleasant over the lifetime (just exactly what the ratio should be is not defined). Subsequent developments of QoL applied to animal welfare [51] have also emphasised the key relationship between QoL and the animal’s subjective experiences. However QoL in humans is seen as a multi-dimensional concept (e.g., [52]) consisting of different domains, suggesting that QoL approaches in animals which are unduly focused on any single aspect or domain may not be inclusive enough to capture accurate assessments of QoL. For example, a recent analysis of QoL studies in dogs and cats receiving chemotherapy [53] found that these studies were mainly focused on assessing the animals’ physical state. Based on QoL approaches for infants, Vols et al. [53] suggest that it is possible to assess animal QoL on a wider range of domains including social and role functioning (see also [54,55]).

As we discussed earlier, there has also been debate over the dimensionality of the motivation–emotion nexus; for example Fraser and Duncan [16] suggested that negative and positive MAS may serve different functions and in that sense be orthogonal to each other. Yeates and Main [2] similarly discuss the relationships between negative and positive emotions and conclude in favour of a single continuum, partly on the basis that positive and negative emotions can mutually inhibit each other (e.g., [54]).

This third feature of PAW leads directly into a discussion about aggregation, i.e., about how to add up different aspects of welfare to a total value. The discussion about this has emerged in connection with attempts to define comprehensive measures of animal welfare at farm or flock level and is also mirrored in Mellor and colleagues’ development of the 5 domains approach to welfare assessment [55].

Traditionally, animal welfare research has focused on applying single welfare indicators, often in an experimental setting, and the question of how to provide a more comprehensive assessment of an animals’ welfare was largely avoided. Since the 1990’s this has changed gradually with the development of systems for assessing overall welfare impact in laboratory animals [56,57] and, since around 2000, initiatives developed to assess farm animal welfare at group level. These initiatives have given rise to more systematic discussions about how to integrate different aspects of what matters to animals (e.g., [58,59]). These efforts have so far culminated in the Welfare Quality^®^ project, that developed protocols to measure the welfare of cattle, pigs and hens at farm level (see [60]).

In these initiatives, a growing understanding has developed of the variety of measures which can be considered to give a comprehensive account of the welfare of animals in a given setting. However, there have only been few attempts to explain and justify, firstly, how to add up these measures to give an account of the net welfare of the affected animals and, secondly, how to draw lines between positive, neutral and negative welfare states. So far the most developed attempt to do this in the Welfare Quality^®^ project has attracted severe criticisms of both a conceptual and an ethical nature [61,62,63,64].

In sum, QoL arguably serves to further convey the importance of considering more than the negative aspects of an animal’s life, highlighting the relevance of assessing the animal’s overall welfare state (inclusive of both negative and positive aspects). However, while the two previously discussed features of PAW—positive emotions and PAE—are both linked to a wider and well established literature, the development of QoL as a key feature of PAW will require more development of the link between PAW and the emerging research agenda on QoL. We see this as particularly concerning: how to derive a comprehensive set of measures covering all aspects of animal welfare; how to add up these measures to give a representation of the net welfare; and, how to draw the lines between positive, neutral and negative net welfare. We will now move onto the final defining feature of PAW, happiness in animals, which is distinct from QoL as being a conception of the animals’ welfare over its lifetime.

### 3.4. Happiness in Animals

Happiness was introduced to PAW by Yeates and Main [2]; indeed they open their paper with reference to a poem by Alexander Pope on human happiness. However they went further when they suggested that there might be animal equivalents to categories of human happiness proposed by positive psychology:

“Human psychologists have advocated dividing the human happiness into (1) ‘the pleasant life’, (2) ‘the engaged life’ and (3) ‘the meaningful life’ [55]. Tentative analogues for animals might be (A) everyday sensational pleasures, (B) engaging with their environment, their conspecifics and their handlers and (C) realising their own goals”.

*([2], p. 296)*

Yeates and Main [2] presented these happiness categories as separate entities, and there is no direct linkage made here between sensations of pleasure and the other categories (e.g., engagement with the environment). However, it is possible to see this idea of happiness as a call for looking at the full life of an animal as a basis for saying that the animal has achieved positive welfare. This contrasts with the literature described in connection with QoL where the set of measures applied to animals typically only aim to look at the state of the animals at a specific point in time. This seems a logical extension of the PAW concept.

#### Happiness in Animals: PAW Contrasted to the Wider Literature

We found that happiness in animals has been discussed and researched in the wider literature, and that the term is used in different ways. For example, one line of work has developed from the study of subjective well-being (SWB) in humans which is also referred to as happiness (e.g., [65]). In a series of papers starting with King and Landau [66], a SWB rating scale developed for humans was modified for zoo keepers to assess happiness in captive primates; this approach has subsequently been applied to captive primates to study the genetics of happiness [67], lifetime changes in happiness [68], and the relationship between happiness and longevity [69]. More recent work has studied the relationship between SWB (happiness) and welfare, again in primates using zoo keepers to assess these attributes on the same animals using different scales [70]. Other experimental work has defined animal happiness as more optimistic performance in a judgement bias test [71]. More recently Webb et al. [72] have reviewed animal happiness integrating human and animal research and concluded that animal happiness can be conceived of as a long-term relatively stable trait that reflects the balance of negative and positive emotions summarised as ‘how an animal feels most of the time’.

The notion of happiness could bring the lived life of each individual animal back into focus when it comes to discussions about PAW, including so far undiscussed aspects such as how experiences across an animal’s life (including during early life) may affect PAW by affecting the ability of animals to make the most of the available opportunities (referred to as live-ability by Webb et al. [64]).

In summary, as with QoL the study of animal happiness is much less established than the study of positive emotions and PAE. Here the idea of PAW may help to inspire an emerging research agenda where the focus is on how events across an animals’ different life phases must interact for animals to be genuinely happy (i.e., to achieve a well-rounded life).

As we have discussed in the preceding sections, PAW can be characterised by four key features; positive emotions, positive affective engagement, Quality of Life and happiness, which vary in their overlap with the wider animal welfare literature. As such, although we would argue that the core elements of PAW are not distinct from the wider animal welfare literature, what the PAW literature has perhaps done is create a space where these elements are more clearly exemplified and, by doing so, made more manifest the importance of looking at the positive end of the scale in the context of animal welfare.

In the following section, we focus more closely on the interconnections between the core elements of PAW and the wider animal welfare literature.

### 3.5. Interconnections between Features of PAW and the Wider Literature

**Figure 1:**
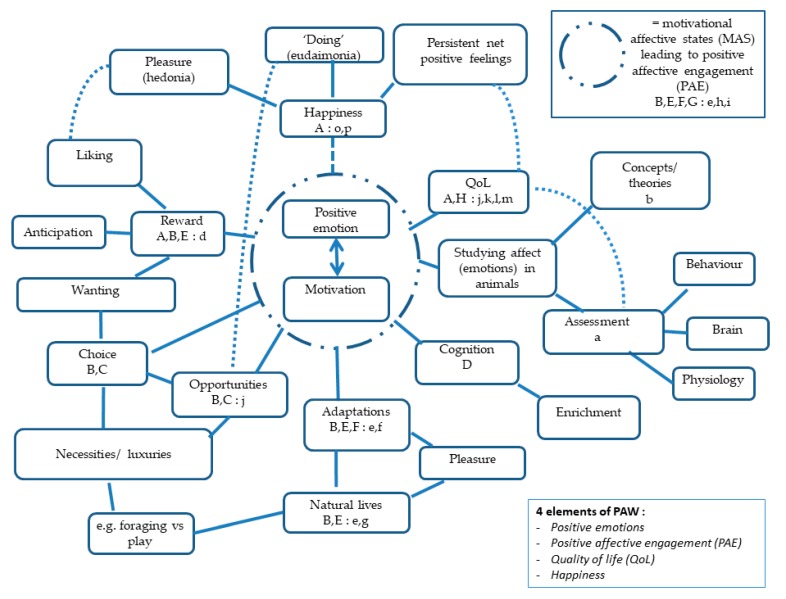
*An interpretation of the inter-relationships between the defining elements of PAW and the wider literature.* Reference lettering is taken from Table 1 and distinguishes between the core PAW and wider literature. Top right box: provides references to the motivation–emotion nexus; Bottom right box: lists the 4 defining elements. Solid lines between terms indicates a definable link has been made in the literature; dotted lines propose potential links/overlaps. We have not included the dotted line linking the pleasure from performing natural behavior to liking and happiness pleasure as this interferes with the readability of the figure.

Our review of the PAW literature has revealed that, despite consisting of only 10 pieces of work, it is complex with a number of features and links to the wider literature. In order to better understand this complexity we have developed a qualitative interpretation of how the different elements of PAW interrelate and link to the wider literature (Figure 1). The following points explain how we arrived at this interpretation: (see Figure 1 legend for explanation of the notation used): (here in the text we cross-refer to papers from the main reference list as [1,2,3,4,5,6,7,8,9,10,11,12,13,14,15,16,17,18,19,20,21,22,23,24,25,26,27,28,29,30,31,32,33,34,35,36,37,38,39,40,41,42,43,44,45,46,47,48,49,50,51,52,53,54,55,56,57,58,59,60,61,62,63,64,65,66,67,68,69,70,71,72,73,74,75,76] with the notation used in Table 1 (A, B, C, D, E, F, G, H, I, J) and (a, b, c, d, e, f, g, h, i, j, k, l, m, n, o, p) (Note: Figure 1 only uses the notation from Table 1):

I. Motivation–emotion: we have placed the motivation–emotion nexus as central to PAW (in effect combining the features of *positive emotions* and *positive affective engagement*) because this resulted in the most optimal arrangement of links between PAW elements and with the wider literature. We also agree with the proposal that animals have evolved a close and intricate relationship between goal-directed behaviour and emotion to better adapt and fulfil their evolutionary goals (e.g., [22] (B)). We would also further propose in a natural state such is the close relationship between motivation and emotion that they can in effect be regarded as a single complex or entity (as proposed by Fraser and Duncan [16] (e)); this approach also avoids debate over the primacy of motivation or emotion ([3] (J)). Note: as indicated in the right hand corner box we have assumed an overlap between the motivation-emotion nexus and the concepts of ‘effectiveness in animals’ ([37] (h)) and ‘animal agency’ ([39] (i)).

Moving clockwise around the Figure starting at the bottom with:

II. Adaptations: Mellor (e.g., [22] (B)) and Fraser and Duncan ([16] (e)) both see the neural components underlying motivation and associated emotions as ‘genetic pre-adaptations’; this links with both affective neuroscience (e.g., Panksepp ([23] (f)), Berridge and colleagues (e.g., [28] (d)), and also the wider animal welfare literature that has long had an interest in animals living ‘natural lives’ and being able to ‘perform most normal behaviour’ (e.g., [42] (g)). Note: both Fraser and Duncan ([16] (e)) and Bracke and Hopster ([42] (g)) make specific reference to the pleasure associated with performance of specific natural (or normal) behaviours.
III. Choice: choice has been another major theme of the wider animal welfare literature (e.g., as a means of assessing ‘behavioural needs’ [17]) and clearly links to the motivation–emotion nexus. Choice also features in the core PAW literature ([9] (C)) and is part of the rationale for the concept of giving animals ‘good life opportunities’ (e.g., [5] (j)). As we indicated earlier, one of the features of good life opportunities is that they are aimed at facilitating both necessities (e.g., foraging when hungry) and luxuries (e.g., play behavior) ([9] (C)).
IV. Reward: reward is an important aspect of the core PAW literature with both Yeates and Main ([2] (A) and Mellor (e.g., [22] (B) discussing the implications of the work of Berridge and colleagues (e.g., [28] (d)), which suggests a distinction between the psychological components of reward in terms of their underlying neural substrates. ‘Wanting’ (or the motivational drive involving incentive salience) is generated by a substantial and distributed brain system. ‘Liking’ (or the pleasure component of reward) is generated by a small number of ‘hedonic hot spots’. Even across very small distances within the same brain centre, different behavioural responses indicative of wanting and liking can be generated (e.g., [28] (d)). This work draws a potential distinction between motivation and emotional responses to stimuli in terms of how the brain generates these sensations; however, as pointed out by Yeates and Main ([2] (A)), this distinction may rarely be relevant under ‘normal’ conditions. Note: there seems to be a clear link between the neuropsychology literature on wanting and the motivationally based use of choice and opportunities in PAW.
V. Happiness: happiness is one of the defining features of PAW and was introduced by Yeates and Main ([2] (A)) in referring to the work of human based positive psychologists such as Seligman ([55] (o)). Happiness in animals has also been reviewed recently by Webb et al. ([72] (p)) somewhat separately to PAW. Human happiness is often distinguished into the categories of pleasure (hedonic happiness) and meaning or purpose (eudaimonic happiness); Yeates and Main ([2] (A)) consider what might be animal equivalents to these separate human-based happiness categories. Note: We have introduced the term ‘doing’ as a more relevant animal-based term to cover eudaimonic happiness. We propose that the pleasure aspects of happiness, the liking component of reward and the pleasure associated with performing normal behavior are all strongly overlapping. Webb et al. ([72] (p)) introduce the concept of affective happiness which can be thought of as how an animal feels most of the time and this we propose overlaps with this conception of happiness and the application of the QoL concept to PAW. We propose that happiness is distinct from QoL, by drawing attention to the whole of the lived life of the animal and how experiences at different life stages can influence how much the animal is able to have a rounded and positive life.
VI. Quality of life:*QoL* is another defining PAW feature largely because of the FAWC (2009) report ([5] (j)) that introduces the idea of a ‘good life’ (as the upper band of a QoL scale for farm animals). QoL is also discussed in the context of PAW by Yeates and Main ([2] (A)) and Mellor ([73] (H)). However we found QoL in the context of PAW to be the most confusing element. FAWC ([5] (j)) do not specifically refer to PAW although it is reasonable to assume that a good life refers to a PAW state. QoL applied to PAW appears to be largely based on the animal’s emotional state (see both ([2] (A)) and ([73] (H)) whereas QoL is more usually seen as consisting of domains (one of which relates to emotional state) (e.g., [53] (m)). Furthermore within PAW there is the development of the Five Domains model of welfare assessment ([73](H)) which for us could also be described as a QoL assessment approach. Therefore, we see a need to develop QoL in the context of PAW by developing approaches to allow different welfare aspects (negative and positive) to be integrated and to place an animals’ welfare on a scale from bad to excellent.
VII. The study of affect (emotions) in animals: this is a substantial field of work that we only refer to through key linking references. We distinguish between research on theories and concepts of affect/ emotions in animals (e.g., [11] (b)) and the assessment of affect/ emotions in animals (e.g., [1] (a)). Interestingly the most direct referencing to PAW can be found in the assessment literature and especially ([1] (a)) which makes several references to the motivation–emotion nexus.
VIII. Cognition: This is the first time we have specifically referred to cognition as we found it not to be a substantial defining feature of PAW. It is referred to in passing by Mellor (e.g., [22] (E)) and by ([74] (D)) and ([75] (I)) in the context of environmental enrichment strategies.

## 4. Discussion

We undertook this review of PAW to better understand how the PAW concept has developed since it was first introduced and also to critically assess how PAW interrelates with and adds to the wider animal welfare literature. Our review shows clearly how small the specific PAW literature is (defined as those papers and book chapters that refer to PAW and clearly develop the concept); it is also the case that of the 10 papers that we identified as core PAW papers, 5 of these are the work of Mellor and colleagues. At the same time, our review illustrates how relatively complex PAW is, being constituted of a number of concepts and ideas emerging from different fields. We found the 4 features of positive emotions, positive affective engagement (PAE), quality of life (QoL) and happiness to define the current PAW literature. We found little evidence to suggest that PAW represents a ‘step-change’ in thinking but is better seen as an evolution from the wider animal welfare literature. Indeed the distinction between the PAW literature and wider animal welfare literature is somewhat arbitrary; in our case being dependent on our search methodology and whether the paper specifically referred to PAW in the title, abstract or author’s keywords. As a result we distinguish between the core PAW literature and papers that best linked the PAW literature to wider, relevant areas of research that did not specifically refer to positive welfare (Table 1).

Among the defining features, we noted the centrality of positive emotions to the development of PAW and discussed various possibilities for why this is, including that for some the growing evidence for positive emotions in animals provides the necessary justification for PAW (e.g., [1,2]). Despite the logic of this position we also see risks in too closely defining PAW through positive emotions including that such a focus reduces attention to other important inputs to overall welfare; indeed it begs the question of how positive emotions emerge in the first place. In our synthesis of the interrelationships between PAW and related concepts (see Figure 1), we propose the emotion–motivation nexus (PAE, in brief) as central because it provides both a link between goal-directed behaviours and the emergence of positive emotions, and also provides links between PAW and the wider literature. As examples, PAE clearly overlaps with the happiness components of doing and the resulting experiencing of pleasure; PAE also links to QoL through the FAWC proposal to provide animals with good life opportunities, which effectively is about providing animals with opportunities for PAE [5,9]. We acknowledge that there may be other ways of visualising these interconnections.

Our review has clearly identified the significant overlap between the concepts and ideas that have variously contributed to PAW (see also Figure 1). PAE, for example, overlaps with existing writings on performing natural (or normal) behaviours (e.g., [42]), motivational affective states (MAS) [16], effectiveness [37] and animal agency (e.g., [38]), all of which point to the close relationships between initiating and completing goal-directed behaviours and positive emotions. PAE also overlaps with ideas of giving animals choices and providing good life opportunities with which to express ‘positive’ goal-directed behaviours [9], and with the study of reward in animals which attempts to disaggregate the motivational and positive emotional aspects of reward seeking behavior (e.g., [28]). As we propose above there are overlaps between the motivation–emotion nexus and happiness, given that happiness in animals can be seen as involving the achieving of goals (doing) and the resulting pleasure (feeling good) [2].

In our view the overlaps between various ideas about how PAW relates to longer-term welfare are less clear because here PAW interacts with only recently emerging research agendas in the study of animal welfare focusing on how to aggregate welfare both at a specific moment of time and across and individual animal’s life (e.g., [61]). In this context the idea of animal happiness adds the important time dimension to PAW but otherwise seems closely overlapping with QoL. Indeed happiness and QoL could be combined if we accept that for the individual animal, it is what is experienced over the longer-term which sums up the negatives and positives of life (see also [55]). Integrating happiness with QoL in this way would bring the lived life of each individual animal into focus when it comes to discussions about PAW. As we discussed earlier, an important discussion here is about how animals’ experiences during different life phases may affect their future PAW or animals’ ability to make the best out of the opportunities they are given (see also [72]).

We have argued that the 4 defining features of the PAW literature are to varying degrees extensions of the wider animal welfare literature and in that sense PAW seems to be a natural development of animal welfare rather than a ‘step-change’. However, it also seems reasonable to us to propose that PAW could be an effective route to changing attitudes to animals and to farming [3], particularly if clear consideration is first given to how key stakeholders in society interpret PAW and how this can affect their response to it [76].

## 5. Conclusions

Our analysis of the PAW literature suggests that it can be defined by 4 features (positive emotions, positive affective engagement (PAE), Quality of Life (QoL) and happiness). The first two features (positive emotions and PAE (or the reward from completing complex motivated behaviours)) are already part of welfare research going back decades. However, the two latter features (QoL and happiness) link to more recent research agendas concerning how we integrate or add up a wide range of welfare measures, and how specific life events may affect the ability of animals to make the most of their life opportunities. Overall, similar to the arguments that are made over positive human psychology, PAW emphasises the capacity for animals to live good lives which in turn could inspire higher aspirations for animal welfare standards.

## Figures and Tables

**Table 1:** (**a**) A chronologically organized list of the core positive animal welfare (PAW) literature; (**b**) a list of literature that links to PAW organized chronologically under the 4 features: positive emotions; positive affective engagement; quality of Life; happiness. Explanations for the inclusion criteria for these lists are contained in the Table. The lettering for this table is used in Figure 1 and cross-referenced against the main reference list in Section 3.5.

(**a**). Core PAW literature: Work that specifically refers to positive animal welfare and clearly contributes to the development of the concept (in chronological order):
Yeates, J.W.; Main, D.C. Assessment of positive welfare: A review. *Vet. J.* **2008**, *175*, 293–300. Mellor, D.J. Animal emotions, behaviour and the promotion of positive welfare states. *N. Z. Vet. J.* **2012**, *60*, 1–8. Edgar, J.; Mullan, S.; Pritchard, J.; Mcfarlane, U.; Main, D. Towards a ‘good life’ for farm animals: Development of a resource tier framework to achieve positive welfare for laying hens. *Animals* **2013**, *3*, 584–605. Boissy, A.; Erhard, H.W. How studying interactions between animal emotions, cognition, and personality can contribute to improve farm animal welfare. In *Genetics and the Behavior of Domestic Animal*; Academic Press: London, UK, 2014; pp. 81–113. Mellor, D.J. Enhancing animal welfare by creating opportunities for positive affective engagement. *N. Z. Vet. J.* **2015**, *63*, 3–8. Mellor, D.J. Positive animal welfare states and encouraging environment-focused and animal-to-animal interactive behaviours. *N. Z. Vet. J.* **2015**, *63*, 9–16. Mellor, D.J. Positive animal welfare states and reference standards for welfare assessment. *N. Z. Vet. J.* **2015**, *63*, 17–23. Mellor, D.J.; Beausoleil, N.J. Extending the ‘Five Domains’ model for animal welfare assessment to incorporate positive welfare states. *Anim. Welf.* **2015**, *24*, 241–253. Krebs, B.; Marrin, D.; Phelps, A.; Krol, L.; Watters, J. Managing aged animals in zoos to promote positive welfare: A review and future directions. *Animals* **2018**, *8*, 116. Lawrence, A.B.; Newberry, R.C.; Špinka, M. Positive welfare: What does it add to the debate over pig welfare? In *Advances in Pig Welfare*; Woodhead Publishing: Duxford, UK, 2018; pp. 415–444.
(**b**). Literature that clearly links PAW to the general animal welfare literature and also to wider science. To be included here papers or reports were required to link substantially and clearly to one of the 4 elements of PAW which we have identified. Work was not required to refer to PAW or to contribute to the PAW concept. We did not seek to provide a comprehensive list of relevant work but to reference what in our judgement were up to five key linking papers for each of the elements (in chronological order):
Positive emotions: (a) Boissy, A.; Manteuffel, G.; Jensen, M.B.; Moe, R.O.; Spruijt, B.; Keeling, L.J.; Winckler, C.; Forkman, B.; Dimitrov, I.; Langbein, J.; et al. Assessment of positive emotions in animals to improve their welfare. *Physiol. Behav.* **2007**, *92*, 375–397. (b) Mendl, M.; Burman, O.H.; Paul, E.S. An integrative and functional framework for the study of animal emotion and mood. *Proc. R. Soc. B Biol. Sci.* **2010**, *277*, 2895–2904. (c) Burgdorf, J.; Panksepp, J. The neurobiology of positive emotions. *Neurosci. Biobehav. Rev.* **2006**, *30*, 173–187. (d) Berridge, K.C.; Kringelbach, M.L. Pleasure systems in the brain. *Neuron* **2015**, *86*, 646–664. Positive affective engagement: (e) Fraser, D.; Duncan, I.J. ‘Pleasures’, ‘pains’ and animal welfare: Toward a natural history of affect. *Anim. Welf.* **1998**, *7*, 383–396. (f) Panksepp, J. Affective consciousness: Core emotional feelings in animals and humans. *Conscious. Cognit.* **2005**, *14*, 30–80. (g) Bracke, M.B.; Hopster, H. Assessing the importance of natural behavior for animal welfare. *J. Agric. Environ. Ethics* **2006**, *19*, 77–89. (h) Franks, B.; Higgins, E.T. Effectiveness in humans and other animals: A common basis for well-being and welfare. In *Advances in Experimental Social Psychology*; Academic Press: San Diego, CA, USA, 2012; pp. 285–346. (i) Špinka, M.; Wemelsfelder, F. Environmental challenge and animal agency. In *Animal Welfare;* Appleby, M., Mench, J., Olsson, A., Hughes, B.O., Eds.; CABI: Wallingford, UK, **2011**, 27–43. Quality of life: (j) Farm Animal Welfare Council. *Farm Animal Welfare in Great Britain: Past, Present and Future*; FAWC: London, UK, 2009; pp. 1–70. (k) McMillan, F.D. Quality of life in animals. Views: Forum. *JAVMA* **2000**, *216*, 1904–1910. (l) Yeates, J. Quality of life and animal behaviour. *Appl. Anim. Behav. Sci.* **2016**, *181*, 19–26. (m) Vøls, K.K.; Heden, M.A.; Kristensen, A.T.; Sandøe, P. Quality of life assessment in dogs and cats receiving chemotherapy—A review of current methods. *Vet. Comp. Oncol.* **2017**, *15*, 684–691. Happiness: (n) King, J.E.; Landau, V.I. Can chimpanzee (*Pan troglodytes*) happiness be estimated by human raters? *J. Res. Personal.* **2003**, *37*, 1–15. (o) Seligman, M.E.; Steen, T.A.; Park, N.; Peterson, C. Positive psychology progress: Empirical validation of interventions. *Am. Psychol.* **2005**, *60*, 410. (p) Webb, L.E.; Veenhoven, R.R.; Harfeld, J.L.J.; Jensen, M.B. What is animal happiness? *Ann. N. Y. Acad. Sci.* **2018**, doi:10.1111/nyas.13983.
